# Supplementary figures and images for: The trisaccharide melezitose impacts honey bees and their intestinal microbiota
Source: PLoS One. 2020 Apr 10;15(4):e0230871. doi: 10.1371/journal.pone.0230871 (PMC7147780; doi:10.1371/journal.pone.0230871)

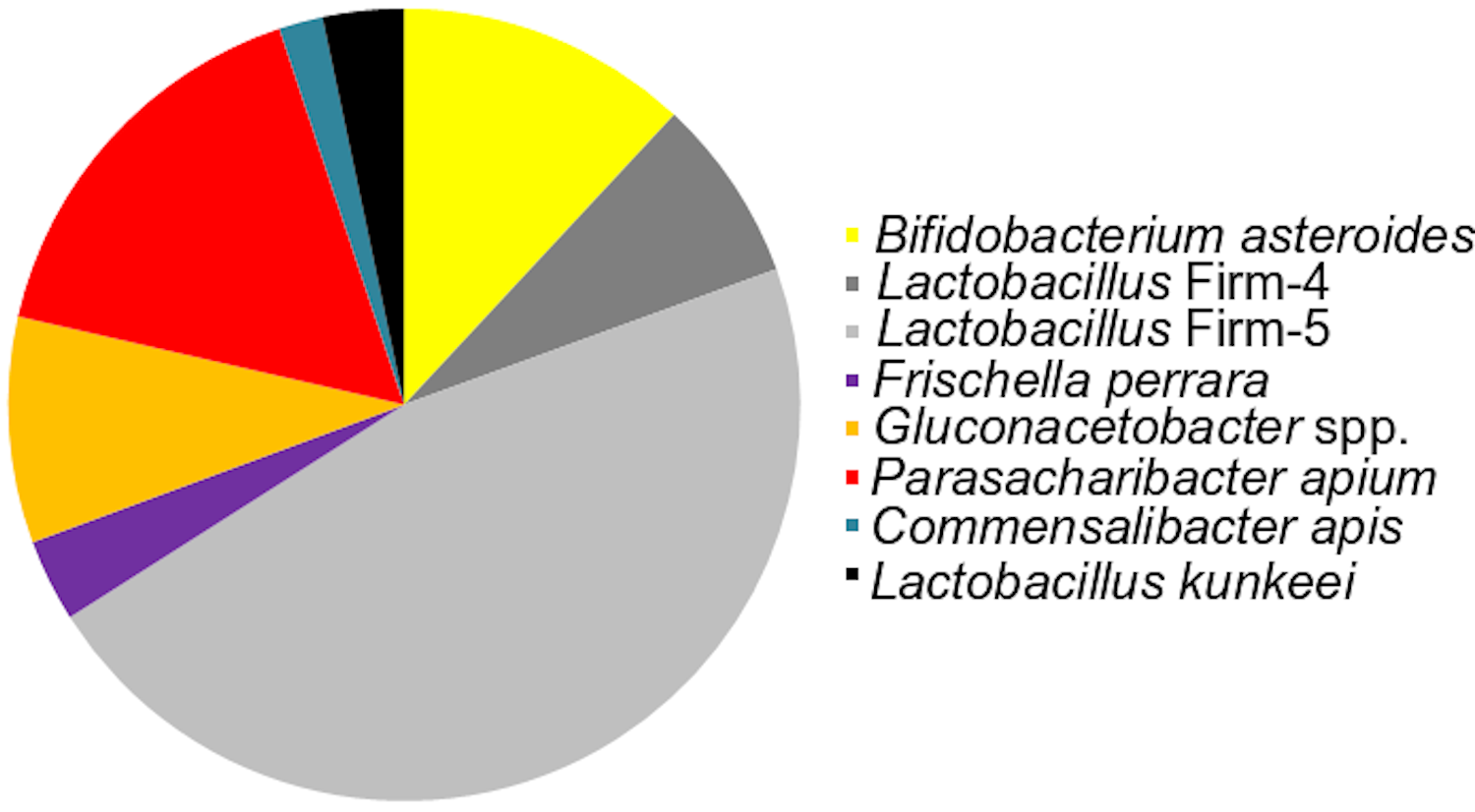

Supplement: S1 Fig — Honey bees were removed from their colonies directly after emerging and had no contact to nurse bees. Bacterial species are highlighted by colour and shown in the legend. Core-members of the honey bee gut microbiota are written in bold. (TIF) [file pone.0230871.s001.tif]

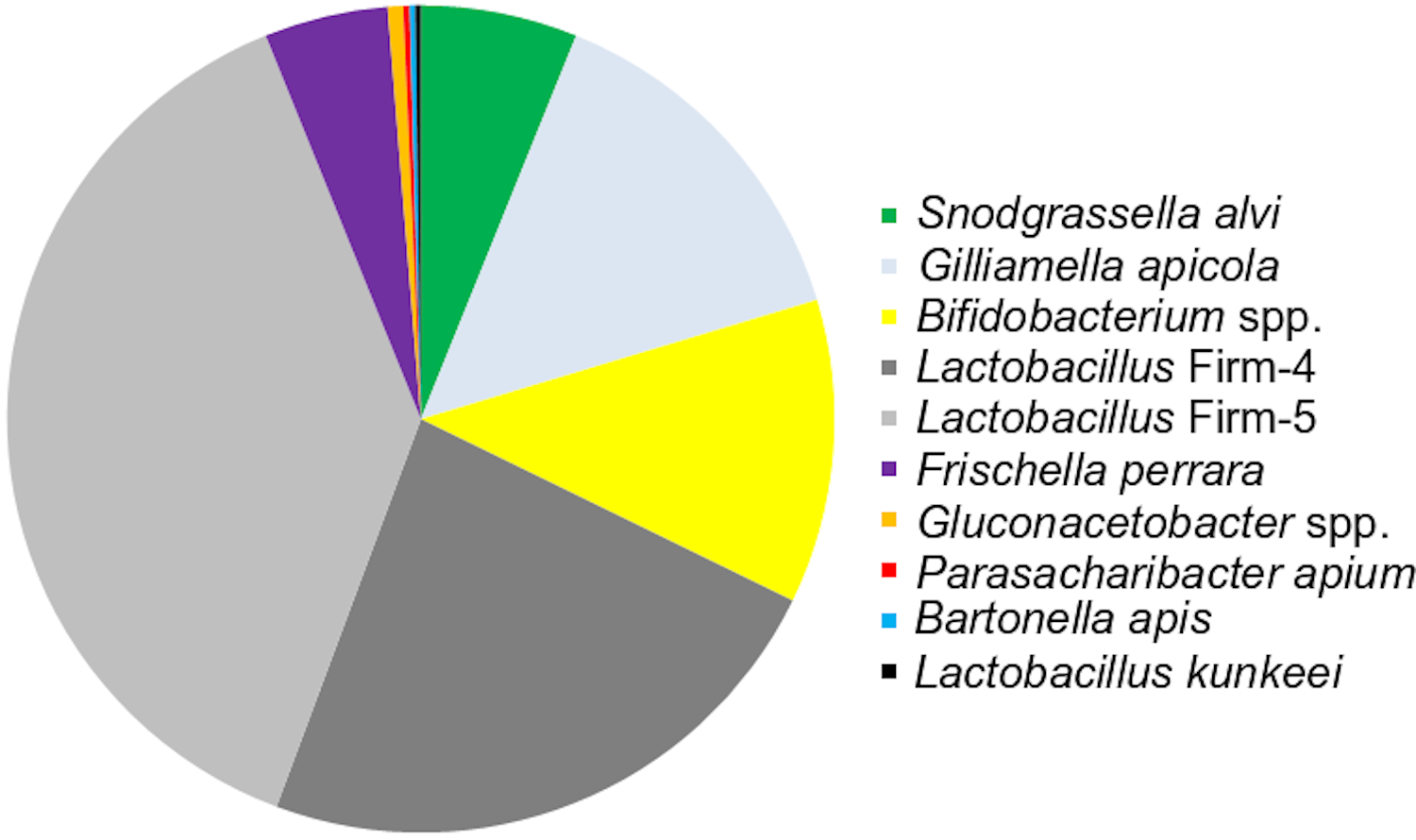

Supplement: S2 Fig — Honey bees lived in bee hive colonies with contact to nurse bees until day 5. Bacterial species are highlighted by colour and shown in the legend. Core-members of the honey bee gut microbiota are written in bold. (TIF) [file pone.0230871.s002.tif]

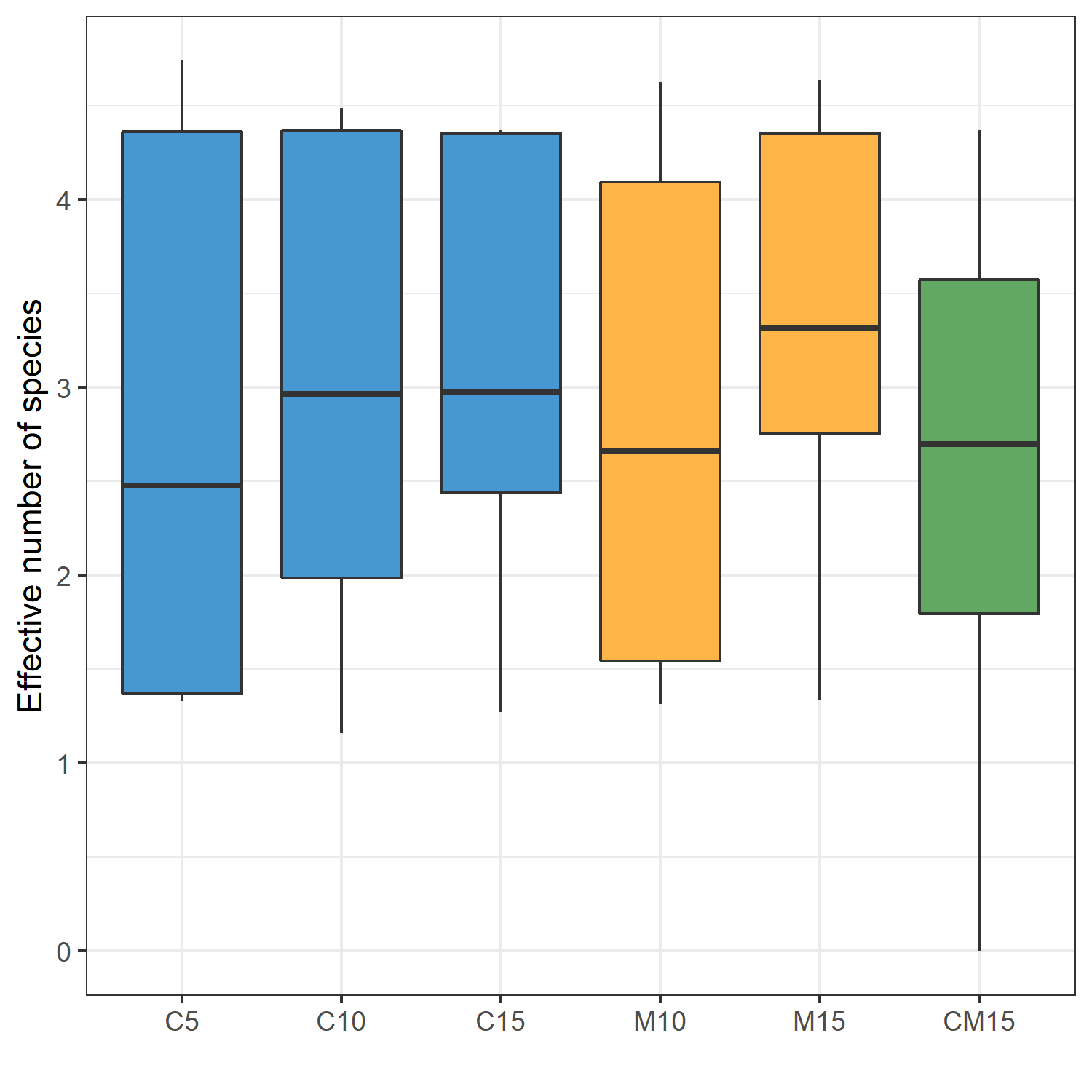

Supplement: S3 Fig — Differences in α-diversity, i.e. Shannon effective number of species, in the gut microbiota of control (blue) and melezitose (yellow) fed bees and bees with a changed diet (green) based on 16S RNA gene amplicon sequencing. No significant differences between the groups were detected (Kruskal-Wallis chi-squared = 1.8162, df = 5, p-value = 0.8739). For each treatment group and honey bee age, 18 honey bee individuals were used for analysis. C = control-fed bees (blue), M = melezitose-fed bees (yellow), CM = bees first fed with control and from day 10 with melezitose (green); 5, 10 and 15 shows the honey bee age in days. The vertical boxplots depict the interquartile range (lower bound/ upper bound of the box correspond to the 25%/ 75% quantile) and the median (horizontal line in the box). (TIFF) [file pone.0230871.s003.tiff]

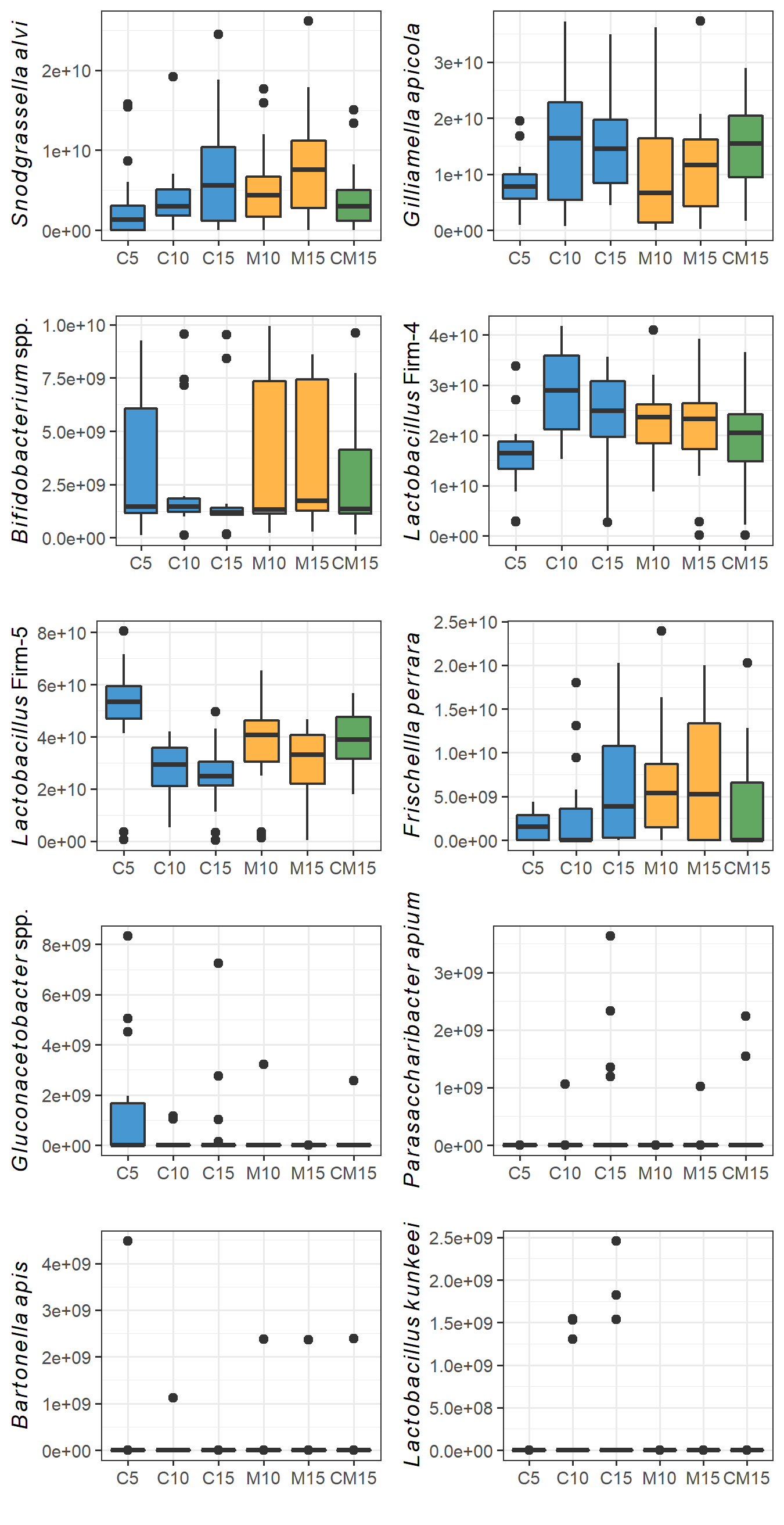

Supplement: S4 Fig — Absolute abundance of the ten monitored phylotypes: Snodgrassella alvi, Gilliamella apicola, Bifidobacterium spp., Lactobacillus Firm-4, Lactobacillus Firm-5, Frischella perrara, Gluconacetobacter spp., Parasaccharibacter apium, Bartonella apis and Lactobacillus kunkeei. The ten plots show the cumulative abundances for each bee. For each treatment group, 18 honey bee individuals were used for analysis. C = control-fed bees (blue), M = melezitose-fed bees (yellow), CM = bees first fed with control and from day 10 with melezitose (green); 5, 10 and 15 shows the honey bee age in days. Significant differences between the treatment groups could be shown for all Lactobacillus species and are demonstrated in Figs 4–6. The vertical boxplots depict the interquartile range (lower bound/ upper bound of the box correspond to the 25%/ 75% quantile), the median (horizontal line in the box) and outlying observations (points outside the box). (TIFF) [file pone.0230871.s004.tiff]
