# Supplementary material for: The trisaccharide melezitose impacts honey bees and their intestinal microbiota
Source: PLoS One. 2020 Apr 10;15(4):e0230871. doi: 10.1371/journal.pone.0230871 (PMC7147780; doi:10.1371/journal.pone.0230871)
Supplement: Attachment — Submitted filename: Response to Reviewers.docx [file pone.0230871.s008.docx]

**PONE-D-19-34528**

**The trisaccharide melezitose impacts honey bees and their intestinal microbiota**

**PLOS ONE**

**Detailed answers:**

To enhance the reproducibility of your results, we recommend that if applicable you deposit your laboratory protocols in protocols.io, where a protocol can be assigned its own identifier (DOI) such that it can be cited independently in the future. For instructions see: [http://journals.plos.org/plosone/s/submission-guidelines#loc-laboratory-protocols](http://journals.plos.org/plosone/s/submission-guidelines#loc-laboratory-protocols)

We were weighing this useful comment but decided to leave the short description of the laboratory work within the manuscript. With this decision, we could fill the manuscript with the missing information of the used reagents as the second reviewer noted.

1. Please ensure that your manuscript meets PLOS ONE's style requirements, including those for file naming. The PLOS ONE style templates can be found at http://www.plosone.org/attachments/PLOSOne_formatting_sample_main_body.pdf and http://www.plosone.org/attachments/PLOSOne_formatting_sample_title_authors_affiliations.pdf

The manuscript has been checked and restructured according to the PLOS ONE’s style requirements.

2. Please include in your methods section the full species name of the bees and also the source of the bees used in this study.

Thank you, we completed the methods section in Line 116-117.

3. We note that you have stated that you will provide repository information for your data at acceptance. Should your manuscript be accepted for publication, we will hold it until you provide the relevant accession numbers or DOIs necessary to access your data. If you wish to make changes to your Data Availability statement, please describe these changes in your cover letter and we will update your Data Availability statement to reflect the information you provide.

We do not wish to make changes to our Data Availability statement.

4. Please remove your figures from within your manuscript file, leaving only the individual TIFF/EPS image files, uploaded separately. These will be automatically included in the reviewers’ PDF.

The figures are removed.

Reviewers' comments:

Reviewer's Responses to Questions

Comments to the Author

1. Is the manuscript technically sound, and do the data support the conclusions?

The manuscript must describe a technically sound piece of scientific research with data that supports the conclusions. Experiments must have been conducted rigorously, with appropriate controls, replication, and sample sizes. The conclusions must be drawn appropriately based on the data presented.

Reviewer #1: Yes

Reviewer #2: Yes

Reviewer #3: Yes

________________________________________

2. Has the statistical analysis been performed appropriately and rigorously?

Reviewer #1: Yes

Reviewer #2: I Don't Know

Reviewer #3: Yes

________________________________________

3. Have the authors made all data underlying the findings in their manuscript fully available?

The PLOS Data policy requires authors to make all data underlying the findings described in their manuscript fully available without restriction, with rare exception (please refer to the Data Availability Statement in the manuscript PDF file). The data should be provided as part of the manuscript or its supporting information, or deposited to a public repository. For example, in addition to summary statistics, the data points behind means, medians and variance measures should be available. If there are restrictions on publicly sharing data—e.g. participant privacy or use of data from a third party—those must be specified.

Reviewer #1: Yes

Reviewer #2: Yes

Reviewer #3: Yes

________________________________________

4. Is the manuscript presented in an intelligible fashion and written in standard English?

PLOS ONE does not copyedit accepted manuscripts, so the language in submitted articles must be clear, correct, and unambiguous. Any typographical or grammatical errors should be corrected at revision, so please note any specific errors here.

Reviewer #1: Yes

Reviewer #2: Yes

Reviewer #3: Yes

________________________________________

5. Review Comments to the Author

Please use the space provided to explain your answers to the questions above. You may also include additional comments for the author, including concerns about dual publication, research ethics, or publication ethics. (Please upload your review as an attachment if it exceeds 20,000 characters)

Reviewer #1: The manuscript by Seeburger et al. describes important experiments on the action of the trisaccharide melezitose on honeybees. In the absence of nectar, honeybees frequently collect honeydew which consists of herbivore excretions. Honey containing honeydew contains large amounts of melezitose and is known to cause malnutrition in overwintering honeybees. The authors performed very interesting feeding experiments with caged bees and show that melezitose-fed bees increased food uptake, had higher gut weighs and elevated mortality compared to control bees. Also, they showed swollen abdomen and impaired movements. Melezitose further changed their lactic acid bacteria community. The authors conclude that melezitose cannot be digested easily and may accumulate in the hindgut, thereby causing severe intestinal symptoms.

The topic of this article is important and exciting for the basic and applied honeybee research community. The manuscript is written very well. Methods and results are explained in a clear way. The statistics are performed correctly. The results are discussed in detail. However, English language could be improved and the authors are advised to have an English native speaker correct their manuscript.

Thank you for your review and comments. We carefully improved the language throughout the manuscript.

Otherwise, I only have a couple of minor points:

1) l. 80 „. . . The concentration of invertase . . . in summer bees and occurs is constantly present in winter bees consistently “; something went wrong with this sentence, please correct.

We corrected this sentence (L. 84).

2) l. 135. Please give a rationale for freezing bees at day 21

The reason is added. We needed to freeze the bees for proofing the ability of honey bees to collect and process the food solution with the different sugar compositions in general (L. 133-135).

3) l. 270, please correct „as was the case”

We changed the phrase (L. 280-281).

4) Fig. 1 Consider using mg instead of g

We changed Fig. 1 accordingly o the comment.

5) Fig. ff. What do the error bars indicate? SEM? STD?

The bars in the boxplot highlight the 25%/75% quantiles (lower bound/ upper bound). They help us to visualise and compare the distributions displayed in Figure 1, 2, 4-6, S3 and S4. We added this information to the figure legends.

6) l .357 “fed with the control diet”

We corrected this sentence (L. 368).

7) l. 386, correct “led to colony losses”

We corrected this sentence (L. 398).

8) l. 387 “beekeepers’ journals”

We corrected this (L. 399).

9) l. 420, correct “died earlier than those fed with . . . .”

We corrected this (L. 444-445).

10) the authors might want to discuss in more detail why wild honeybee colonies in forests (not only in Germany but world-wide) apparently do not suffer as much from honeydew than honeybee colonies maintained by beekeepers. Is there an adaptation to melezitose possible?

Thank you, we discussed this in line 483-492.

Reviewer #2: Overall comments:

1. This is an interesting study, and well-structured, with useful and relevant information and excellent findings. That melezitose influences honey bee health is made very clear, and links to microbiome-mediated changes are being suggested.

Thank you very much for your comments and valuable suggestions.

2. There are very minor English issues, in terms of spelling, grammar, which may be corrected during the editor check. For e.g. ad libitum should be in italics, no need for a “-“ between number and units, consistency of units to SI, centrifugal force g in italics, for the reagents the company name to be followed by city and country, melezitose feds hould have a hyphen in between, spp. not to be in italics, Firm-4 an Firm 5 are usually not in italics., Lactobacillus should always be in italics,

We improved the English language throughout the manuscript and added the missing information.

Detailed comments:

Abstract

Higher molecular weight?

We changed this (L. 20).

Digested by the host?

We also made this change (L.31).

Rephrase 16S-amplicon sequencing?

We rephrased it (L. 28-30).

Introduction

Line 53. Mineral content higher by how much? If it is 2 % it may not matter, if it is 20 % it may.

We noticed a second publication reporting this exactly. The mineral content of honeydew honeys compared to blossom honeys is, dependent on the mineral, up to four times higher (L. 55-58).

Line 187. Why were whole bees used? Why not just the gut pulled out?

We are using the RNA-DNA-Extraction as a standard protocol, where whole bees are used and the RNA and DNA can be extracted simultaneously (L.199-200), suitable for follow-up questions.

Results

Line 285/286. Perhaps delete one of the “on the other hand”…. And rephrase.

We rephrased this (L. 296-298).

Line 305. What is numerically slightly weaker?

The effect of the higher gut-body weight ratio was numerically weaker (six percentage-points increase of gut-body weight ratio instead of nine percentage points increase) for the bees fed with melezitose starting from day ten. We added this to the text (L. 316-318).

Discussion

Lines 394-396. Perhaps rephrase. It is not clear what is being meant here.

We rephrased it (L. 408-411).

Line 414- 418. The shift from the microbiome to a lower Firm-4/L. kunkeei and higher Firm-5 relative abundance may be better explained by describing the metabolic versatility and location of the bacteria that are seen in these two groups. Please refer to https://www.ncbi.nlm.nih.gov/pubmed/24944337 and https://www.ncbi.nlm.nih.gov/pmc/articles/PMC6478020/

And that much better explains your statements in lines 421-423.

Thank you very much for suggesting these references, we included them (L. 432-442).

Line 432. What does “sugar drains the bees“ mean? Not clear.

We rephrased it with ‘dehydration’ (L. 457).

Line 435- “sever problems” is too general. Please describe in terms of viability, or metabolic changes or changes in populations.

We explained that melezitose is poorly utilised by honey bees (L. 459-461).

Line 460. Compensate what problems?

We referred to honey bee diseases in general (L. 516).

Line 465-467. The reason for this assumption is not clear at all.

We rephrased this and added a reference (L. 513-515).

Reviewer #3: The authors Seeburger et al. report melezitose-containing honey effects on honey bees. Melezitose is shown to be the cause for the so called ‘honeydew flow disease'. Three independent feeding experiments with caged bees as well as field bees were examined. Bees fed with melezitose showed intake effects, gut weights effects, and showed a lower survival. Several disease symptoms were recorded in melezitose-fed bees. Melezitose changed the gut microbiota too. The MS is written in a clear and concise manner. The Title and Abstract cover the MS contents. The Introduction gives a strong basis. A need for this study is clear.

This MS gives valuable insight to what extent melezitose evokes honey bee colony disease behaviors. The M&M are clear and described into appropriate depth. The statistics are applied appropriately too. Results are reported clearly, and also properly covered by the Discussion. I think the figures are well selected, regarding placement of many as Supplementary Materials. Reference are good, with no inappropriate citations. The conclusion, that melezitose cannot be easily digested and may accumulate in the hindgut is supported by the results. Accumulated melezitose causes intestinal symptoms from which the bees can die. Overall, I like to complement the authors with this MS which is a pleasure to read.

Thank you for sharing your opinion on our manuscript and the valuable feedback.

________________________________________

6. PLOS authors have the option to publish the peer review history of their article (what does this mean?). If published, this will include your full peer review and any attached files.

If you choose “no”, your identity will remain anonymous but your review may still be made public.

Do you want your identity to be public for this peer review? For information about this choice, including consent withdrawal, please see our Privacy Policy.

Reviewer #1: No

Reviewer #2: No

Reviewer #3: No

[NOTE: If reviewer comments were submitted as an attachment file, they will be attached to this email and accessible via the submission site. Please log into your account, locate the manuscript record, and check for the action link "View Attachments". If this link does not appear, there are no attachment files to be viewed.]

While revising your submission, please upload your figure files to the Preflight Analysis and Conversion Engine (PACE) digital diagnostic tool, https://pacev2.apexcovantage.com/. PACE helps ensure that figures meet PLOS requirements. To use PACE, you must first register as a user. Registration is free. Then, login and navigate to the UPLOAD tab, where you will find detailed instructions on how to use the tool. If you encounter any issues or have any questions when using PACE, please email us at figures@plos.org. Please note that Supporting Information files do not need this step.

________________________________________

In compliance with data protection regulations, you may request that we remove your personal registration details at any time. (Remove my information/details). Please contact the publication office if you have any questions.
